# Comparative and Cost Effectiveness of Telemedicine Versus Telephone Counseling for Smoking Cessation

**DOI:** 10.2196/jmir.3975

**Published:** 2015-05-08

**Authors:** Kimber P Richter, Theresa I Shireman, Edward F Ellerbeck, A Paula Cupertino, Delwyn Catley, Lisa Sanderson Cox, Kristopher J Preacher, Ryan Spaulding, Laura M Mussulman, Niaman Nazir, Jamie J Hunt, Leah Lambart

**Affiliations:** ^1^University of Kansas Medical CenterDepartment of Preventive Medicine and Public HealthKansas City, KSUnited States; ^2^University of Missouri, Kansas CityDepartment of PsychologyKansas City, MOUnited States; ^3^Vanderbilt UniversityDepartment of Psychology & Human DevelopmentNashville, TNUnited States; ^4^University of Kansas Medical CenterCenter for Community EngagementKansas City, KSUnited States; ^5^University of Missouri, Kansas CitySchool of Nursing and Health StudiesKansas City, MOUnited States

**Keywords:** telemedicine, Internet, rural, smoking cessation, RCT, primary care

## Abstract

**Background:**

In rural America, cigarette smoking is prevalent and health care providers lack the time and resources to help smokers quit. Telephone quitlines are important avenues for cessation services in rural areas, but they are poorly integrated with local health care resources.

**Objective:**

The intent of the study was to assess the comparative effectiveness and cost effectiveness of two models for delivering expert tobacco treatment at a distance: telemedicine counseling that was integrated into smokers’ primary care clinics (Integrated Telemedicine—ITM) versus telephone counseling, similar to telephone quitline counseling, delivered to smokers in their homes (Phone).

**Methods:**

Smokers (n=566) were recruited offline from 20 primary care and safety net clinics across Kansas. They were randomly assigned to receive 4 sessions of ITM or 4 sessions of Phone counseling. Patients in ITM received real-time video counseling, similar to Skype, delivered by computer/webcams in clinic exam rooms. Three full-time equivalent trained counselors delivered the counseling. The counseling duration and content was the same in both groups and was available in Spanish or English. Both groups also received identical materials and assistance in selecting and obtaining cessation medications. The primary outcome was verified 7-day point prevalence smoking abstinence at month 12, using an intent-to-treat analysis.

**Results:**

There were no significant baseline differences between groups, and the trial achieved 88% follow-up at 12 months. Verified abstinence at 12 months did not significantly differ between ITM or Phone (9.8%, 27/280 vs 12%, 34/286; *P*=.406). Phone participants completed somewhat more counseling sessions than ITM (mean 2.6, SD 1.5 vs mean 2.4, SD 1.5; *P*=.0837); however, participants in ITM were significantly more likely to use cessation medications than participants in Phone (55.9%, 128/280 vs 46.1%, 107/286; *P*=.03). Compared to Phone participants, ITM participants were significantly more likely to recommend the program to a family member or friend (*P*=.0075). From the combined provider plus participant (societal) perspective, Phone was significantly less costly than ITM. Participants in ITM had to incur time and mileage costs to travel to clinics for ITM sessions. From the provider perspective, counseling costs were similar between ITM (US $45.46, SD 31.50) and Phone (US $49.58, SD 33.35); however, total provider costs varied widely depending on how the clinic space for delivering ITM was valued.

**Conclusions:**

Findings did not support the superiority of ITM over telephone counseling for helping rural patients quit smoking. ITM increased utilization of cessation pharmacotherapy and produced higher participant satisfaction, but Phone counseling was significantly less expensive. Future interventions could combine elements of both approaches to optimize pharmacotherapy utilization, counseling adherence, and satisfaction. Such an approach could commence with a telemedicine-delivered clinic office visit for pharmacotherapy guidance, and continue with telephone or real-time video counseling delivered via mobile phones to flexibly deliver behavioral support to patients where they most need it—in their homes and communities.

**Trial Registration:**

Clinicaltrials.gov NCT00843505; http://clinicaltrials.gov/ct2/show/NCT00843505 (Archived by WebCite at http://www.webcitation.org/6YKSinVZ9).

## Introduction

### Background

Globally, an estimated 1 billion people will die from tobacco-related illnesses this century [1]. Although some progress has been made in driving down the prevalence of tobacco use, in rural areas headway lags. For example, the prevalence of smoking in US rural areas was 26% in 2009—equivalent to overall US smoking rates in 1990 [2]. Despite this, few initiatives focus on helping rural smokers quit.

Rural primary care is a good conduit for cessation efforts. Approximately 70% of smokers visit their health care provider in any given year [3]. Unfortunately, during primary care encounters few receive clear advice to quit, only 1 in 5 identified smokers receive cessation counseling, and less than 5% receive pharmacotherapy [4-6]. Barriers to providing tobacco treatment include constraints on time, lack of counseling skills, and poor office systems for conducting intensive, longitudinal behavior change intervention, such as cessation counseling [7-10].

To work around these barriers, primary care providers are increasingly referring patients to proactive tobacco quitlines. Quitlines are available in nearly all US states and many countries, they reach rural areas, and they are effective for smoking cessation [11]. They are, however, poorly utilized—only 1-2% of smokers in the United States have reported trying the quitline [12]. In addition, quitlines operate parallel to the health care system, and in general provide access to cessation medications to only a small subset of callers who meet criteria. Physician-office delivered telemedicine, as delivered by real-time, two-way video counseling, is another promising system for delivering expert care at a distance.

Telemedicine has been shown to deliver effective care for multiple health behaviors and outcomes [13]. A Cochrane review of telemedicine versus in-person patient care found telemedicine and in-person treatment to be equally effective, and both achieved high levels of satisfaction among patients and providers [14]. To date, the only large-scale study evaluating telemedicine for smoking cessation is a group-based intervention trial conducted by Carlson and colleagues in Canada, which achieved equivalent quit rates between groups receiving in-person versus telemedicine-delivered interventions [15]. However, in this study, participants were not randomized into groups and quit rates were based on self-report.

Our objective in “Connect2Quit” was to determine the comparative effectiveness and cost-effectiveness of two ways of delivering expert cessation services at a distance: telemedicine-delivered counseling, integrated into clinical practices (ITM) and quitline-like telephone counseling (Phone). We also examined participant satisfaction with the two approaches. The study employed a two-arm, individually randomized design that examined the impact of ITM on verified cessation at 12 months post-enrollment. We designed ITM to optimize use of the two cornerstones of evidence-based tobacco treatment: counseling and pharmacotherapy [3]. We also designed ITM to be fully integrated into the patients’ routine clinical care. ITM counselors delivered all sessions in participants’ physician offices—counselors scheduled sessions with clinic receptionists, updated the primary care team on participants’ progress, and worked with rural providers to help participants select and obtain medication prescriptions. Because telemedicine counseling occurred in the medical home, participants had the opportunity to immediately ask their health care providers for additional advice regarding pharmacotherapy and prescriptions for smoking cessation medication.

We hypothesized that ITM would outperform Phone by providing much more comprehensive support than could be achieved by Phone alone. Our ITM intervention was designed to (1) deliver a very high-quality, supportive counseling interaction by creating a more personal bond by enabling counselors to respond to important non-verbal cues during counseling, and (2) remove barriers to high-quality advice on prescription and non-prescription cessation medications by creating multiple opportunities for patients to interact with their health care providers over medication choices. For a detailed description of the study design and underlying theory, please see Mussulman et al, 2014 [16].

### Hypotheses

Our study hypotheses addressed outcomes and costs (Textbox 1). These hypotheses are based on several features of the study design. We co-located video counseling in the doctor’s office in order to enable all rural smokers, even those with no computers or poor access to high-speed Internet, to participate. Co-location also created better access to providers and support in obtaining prescription medications. In addition, the visual connection afforded by ITM could result in better counseling adherence and participant satisfaction. To ensure that outcomes could be attributed to the intervention, and not differences in the content or quality of counseling delivery, we also assessed fidelity to the counseling protocols.

**Study aims and hypotheses.**

First Aim: To test the effects of Integrated Telemedicine (ITM) versus Phone on smoking cessation and other smoking outcomes. Compared to participants in Phone, at 12 months following randomization:

Hypothesis 1: Smokers receiving ITM will have significantly higher 7-day point prevalence abstinence (defined as no cigarettes in the past 7 days, biochemically verified).

Hypothesis 2: Smokers receiving ITM will have significantly higher prolonged abstinence.

Hypothesis 3: Smokers receiving ITM will have participated in more counseling sessions and been more likely to use cessation medications.

Hypothesis 4: Among those who continue to smoke, persons receiving ITM will have more quit attempts and will smoke fewer cigarettes.

Second Aim: To examine the costs and cost-effectiveness of ITM versus Phone.

Hypothesis 5: ITM will be more costly, but more cost-effective than Phone from provider, participant, and societal (combined) perspectives. Relative costs of care will be assessed by examining quit rates for ITM and Phone per combined provider and/or participant costs to assess the cost per quit in the two treatment arms from the three perspectives.

## Methods

### Design and Overview

We employed a control group design with individual randomization to study arms. Study staff screened patients for eligibility, collected informed consent, and administered baseline data collection. The counseling approach, content, and educational materials were the same across both ITM and Phone conditions. Within both treatment arms, all participants received the same educational materials and individually tailored pharmacotherapy guidance to help them select and obtain cessation medications. Patients in ITM received 4 sessions of telemedicine counseling integrated into the patient’s primary care office, in examining rooms equipped with 2-way webcams mounted on desktop computers. Participants assigned to Phone received 4 sessions of in-home telephone counseling. Study assessments were conducted at baseline and months 3, 6, and 12. The University of Kansas Medical Center Ethics Committee approved all study procedures. A detailed description of the study intervention, design, and participant baseline characteristics have been published previously [16].

### Setting

Participants were patients of 20 primary care clinics in the state of Kansas. The clinics were located in a wide range of rural counties, half were in cities with a population of less than 1800, and three were federally-qualified health clinics for the medically underserved. We used the Health Resources and Services Administration (HRSA) guidelines to define rural areas; at the time of the study in Kansas, this included 88 non-metropolitan counties and other regions [17].

### Participant Eligibility

Eligible smokers were required to have a primary care physician who was participating in the study, be 18 years of age or older, smoke 5 or more cigarettes per day for at least 1 year, smoke 25 out of the past 30 days, speak English or Spanish, and have a telephone. We opted to take all smokers willing to participate, regardless of level of motivation to quit, in order to maximize the generalizability of the trial. Individuals were excluded if they used other tobacco products, were currently taking smoking cessation medications or participating in another quit smoking program, were breast feeding, were pregnant or planning to become pregnant, were planning on moving in the next year, or lived with a smoker already enrolled in the study.

### Participant Identification, Recruitment, and Randomization

Patients from clinic sites were recruited on site by clinic staff and via mailings from clinic directors. In order to ensure adequate representation of Latino patients, study staff conducted community-based recruitment activities through radio interviews, health fairs, community newsletters, and staff recruitment tables at Latino worksites, religious organizations, and businesses. During on-site recruitment, clinic staff or medical student volunteers identified smokers, screened for eligibility, invited smokers to participate, and sent participant information to study staff who collected informed consent and baseline study data. In recruitment via mailings, letters from clinic leaders informed patients that they would be contacted about a research opportunity from study staff; the letters also provided a number for patients to call in order to proactively opt in or opt out of the trial. Study staff called all smokers and performed screening, consent, and baseline data collection. The project director allocated enrolled participants to study arm by opening sealed envelopes that contained randomly generated group assignments created in advance by the study statistician (KJP) and database manager (NN). Participants were recruited from June 2009 through June 2011.

### Equipment and Site Orientation

Video counseling was delivered via Polycom PVX, a program installed on desktop computers and linked to the University of Kansas Medical Center study staff via the Internet. Each participating site received a desktop computer, webcam, and Polycom PVX software. A study technician installed equipment, tested connections with the site delivering the intervention, and trained clinic staff in equipment use and troubleshooting. The technician placed a binder with connection checklists, troubleshooting tips, and emergency phone numbers next to the study equipment. The technician also met with Internet service managers at each site to set up lines of communication for problem-solving connection issues that might arise throughout the trial. Once equipment was installed, the study project director conducted clinic staff training with each site via the Polycom system, in order to reinforce skills and build confidence in using the system. During this meeting, the project director reviewed study materials with the clinic staff, focusing on the clinic role in care such as reviewing prescription requests and providing medication prescriptions, as outlined below.

### Interventions

#### Overview

Within the first week after enrollment, all study participants received a mailed packet of study materials. The packet included educational materials on smoking cessation and a timeline of study activities, including counseling sessions and follow-up.

The packet also included a pharmacotherapy guidance form, which provided individually tailored information on what medications were covered by the participants’ insurance plan or public assistance program. The guidance form also indicated for what medications patients were medically eligible. Medical eligibility was ascertained by a study pharmacist by entering participants’ prescription medication use and pre-existing health conditions into a pharmacy database to identify contraindications and cautions for each cessation medication. Counselors then called participants to advise them of their group assignment and to schedule the first counseling session. In both study conditions, study staff assisted income-eligible participants with no insurance coverage to apply for cessation medication from the pharmacy assistance programs (PAPs) of pharmaceutical drug companies. Study staff worked with participants and providers to complete these forms and apply to companies for medications.

The counseling approach used across both conditions—ITM and Phone—was based on Combined Behavioral Intervention (CBI), a combination of Motivational Interviewing and Cognitive Behavior Therapy (CBT) [18-20]. The counseling content was designed to adapt to smokers’ level of motivation. The first session included assessment of participants’ readiness to quit, motivational counseling among those not ready to quit, and development of a quit plan among those ready to quit. As part of the quit plan, counselors reviewed participants’ pharmacotherapy options and helped participants select and obtain a cessation medication to aid in quitting. In subsequent sessions, counselors reviewed participants’ progress, helped troubleshoot difficulties, and, time permitting, invited participants to choose a topic for discussion from a list of common barriers to cessation such as “triggers” for smoking or avoiding weight gain. Three full-time equivalent trained counselors delivered the counseling. Prior to each counseling session, counselors telephoned participants to remind them of the session.

#### Integrated Telemedicine (ITM)

Participants in ITM received 4 sessions of clinic-based video telemedicine counseling for smoking cessation. Because most ITM computers were located in dedicated rooms in study clinics, participants could sign in at the clinic reception and go directly to the ITM room for their session. Clinic staff, either a receptionist or a nurse, called the study counselor at the medical center via Polycom PVX to initiate the session. At the close of the counseling session, study counselors directed participants to go to the clinic receptionist. Counselors then telephoned the front desk to schedule the next appointment with the participant and receptionist. If the participant created a quit plan and/or expressed interest in pharmacotherapy, the quit plan and a medication prescription request form were faxed to the receptionist for placement in the participants’ medical record and for review/prescription approval by the participants’ primary care provider.

#### Phone (Telephone Counseling)

Participants in Phone received 4 counseling sessions via their home or mobile phones. At the end of each session, counselors scheduled the next counseling session with the participant. If the participant created a quit plan and/or expressed interest in pharmacotherapy, the quit plan and a medication prescription request form were mailed to the participant, with instructions to take the forms to their health care provider for placement in their medical records and review/prescription approval by their primary care providers.

### Data Collection and Reimbursement

All assessments were conducted via telephone and mail by trained study staff. Assessments occurred at the following times and were reimbursed (US $) at the following levels: baseline ($20), 3 months ($20), 6 months ($20), and 12 months ($50). Clinics that participated in the study received a $1000 reimbursement for incidental costs associated with the trial. In addition, intervention sites received a computer and Polycom PVX software used to implement the intervention. Clinics dedicated the equipment to the telemedicine trial for the duration of the study but kept the equipment at the end of the trial. Prior to the 6- and 12-month follow-up assessments, participants received reminder postcards.

### Measures

#### Baseline Characteristics and Computer/Telemedicine Use

We collected general demographic variables such as age, gender, marital status, education, employment status, income, race, and ethnicity. Smoking history included number of cigarettes per day, quitting history, previous quit smoking medication use, and age of smoking initiation. Nicotine dependence was assessed with the 6-item Fagerström Test for Nicotine Dependence scale (FTND) [21]. Stage of behavioral change was determined using a 4-item algorithm that defines pre-contemplation as having no interest in quitting in the next 6 months, contemplation as strong interest in quitting in the next 6 months, and preparation as strong interest in quitting in the next month coupled with a serious quit attempt in the past year [22]. Motivation and confidence to quit smoking were measured using 10-point Likert scales with higher scores indicating greater motivation and confidence [23]. We summarized income into a dichotomous variable of whether or not income was less than 200% of the 2009 US Federal Poverty guideline.

Participants were also asked four questions related to their perceptions of using computer technology such as telemedicine for the delivery of health care [16]. Computer and Internet availability within the home were also assessed [16].

#### Intervention Fidelity

To assess whether counseling was the same across ITM and Phone sessions, we obtained independent ratings of counselor adherence on a 10% randomly selected subset of sessions. These audio files were encrypted, blinded regarding group assignment, and emailed to an independent expert rater for evaluation via the Motivational Interviewing Treatment Integrity (MITI) coding system [24]. Four variables from this coding system were used to compute a score of adherence to counseling style: (1) Empathy, in which coders assigned a global rating of empathy to the counselor’s style), (2) Spirit, in which coders assigned a global rating of MI spirit to the counselor’s style, (3) MI adherent, a sum of the frequency MI adherent utterances, and (4) MI non-adherent, similarly, a sum of the frequency non-MI adherent utterances. To test for differences between groups on each of these variables, we took clustering (both within participant and within counselor) into account using the multilevel regression module available in SPSS (MIXED) [25].

#### Hypothesis 1: Primary Outcome

The main outcome measure was verified 7-day point prevalence smoking cessation at 12 months. Abstinence verification was assessed via salivary cotinine, carbon monoxide (CO), or proxy. All participants who self-reported being abstinent from cigarettes for the 7 days preceding their 12-month survey, and who were not taking nicotine replacement therapy, were asked to provide a mailed salivary cotinine sample, for which they were reimbursed an additional $50. To reduce any incentive to misreport smoking status, participants were not informed of the $50 additional incentive for verification until after they completed the 12-month questionnaire. Participants meeting the recommended salivary cotinine cut point of <15 ng/ml were considered abstinent [26]. Participants reporting abstinence who were taking nicotine replacement were asked to meet staff at the clinic or at a community location to provide an expired CO (carbon monoxide) sample. Participants meeting the recommended CO cut point of <10 ppm were considered abstinent [27]. Staff contacted proxies to verify abstinence among participants who did not provide cotinine or carbon monoxide. All participants who failed to verify abstinence were counted as smokers. To explore group differences in outcome throughout the follow-up period, we also report self-reported quit rates at months 3, 6, and 12.

#### Hypotheses 2-3: Prolonged Abstinence, Counseling Adherence, Pharmacotherapy Use

Prolonged abstinence as defined in this study included a “grace period” of 1 month at the beginning of treatment to allow the treatment to take effect followed by continuous abstinence [27]. Counseling adherence was collected from counselor records of completed sessions. Pharmacotherapy use was collected via participant self-reports of any prescription or non-prescription use at any time between baseline and follow up.

#### Hypothesis 4: Quit Attempts and Number of Cigarettes Smoked Among Continued Smokers

Quit attempts were assessed by self-report of the number of times patients tried to quit for 24 hours or more since the beginning of the study. Cigarettes per day were self-reported by participants who continued to smoke at month 12.

#### Hypothesis 5: Costs

After itemizing the resources needed for each arm of the intervention, we selected only those items that differed between treatment arms for the variable cost analysis. The fixed costs stemming from the Polycom PVX technology were not included in the cost analysis, consistent with current guidelines for short-run cost analyses. We included costs from both the provider and participant perspectives. All costs were calculated based on 2011 dollars. Since the intervention was completed in less than one year, no discounting was applied. While there may have been limited price inflation during the time period of study recruitment, we did not adjust for inflation that might occur with recruitment of subjects across different years. We used two-tailed *t* tests to examine differences in costs.

### Provider’s Perspective

From the provider’s perspective, a major potential cost difference was counselor’s time. Counselors’ time was summed across the intervention sessions (the date and time of individual sessions were recorded in a database by study staff) and valued at the median national wage plus 25% fringe rate for a health educator (occupation code 21-1091) from the Bureau of Labor Statistics National Occupational Employment and Wage Estimates [28].

For the Phone arm, the communication between counselor and study participant took place over the telephone: we used our local, institutional phone charge rate (US $0.0355/minute). For the ITM arm, communication occurred via the Internet: we collected data from Internet providers in each of the clinic locations on average monthly charges. These charges were converted to hourly rates assuming the clinics were open 9 hours per day, 5 days per week. Given that most clinics may have multiple computers with Internet access, this rate likely overstates the actual cost of Internet-based communication (US $0.37/hour=$0.0062/minute).

Finally, the need for office space to deliver ITM incurred space allocation costs not encountered in the Phone arm. Generally, in cost analyses, space is valued on the basis of opportunity—what the space is normally used for when it is not being used for the intervention being evaluated. In sites where telemedicine was delivered in examining rooms, it should rightfully be valued as the cost of a medical visit. However, some of our sites used other space, either administrative or even storage space, for telemedicine visits. A few, based on changing needs of the clinic, moved telemedicine equipment between administrative and examining room space. We were not always able to determine the space used for every telemedicine visit.

In order to estimate the costs of the space under these varying scenarios, we made two quite different assumptions in assigning a value for this space. First, a functioning exam room could be used to generate billable physician services: therefore, we applied the American Medical Association’s CPT (Current Procedural Terminology) rates for patient visits based upon the length of the visit and 2011 Medicare rates for the facility charges in Kansas (CPT codes=99211, <5 minutes; 99212, 5-10 minutes; 99213, 10-15 minutes; 99214, 15-25 minutes; 99215, 25+ minutes) [29].

Second, for a more conservative estimate, we assumed that the ITM could be delivered in a more general office space; to arrive at this value, we applied local rent costs (US $). Since all sites were rural, we used a rent plus maintenance/utility rate of $10/square foot per year and measured the square footage for each of the clinic sites (mean 148 sq. ft.). We then applied an average rent charge per minute of counseling ($0.0105/min).

### Participants’ Perspective

To capture the participants’ perspective, we valued their time spent in counseling, travel time and costs, and the cost of pharmacotherapy, if applicable. Participants were asked to provide their hourly wage rate: for those who did not, we used age- and gender-adjusted wage rates (minimum wage=US $7.25) from the 2007 Bureau of Labor Statistics [28]. These rates were applied to counseling time. In addition, participants in ITM incurred costs travelling to and from clinic offices for sessions that were not incurred by Phone participants. Travel time was calculated based upon the distance from the participant’s residence to and from the clinic site for the ITM arm using Google maps (maps.google.com). Mileage costs were added as vehicle costs using the state-based reimbursement rate for travel (US $0.54/mile). Finally, we collected self-reported (out-of-pocket) pharmacotherapy costs from participants.

### Societal Perspective and Cost Per Quit

We calculated the society perspective on costs by summing provider and participant perspectives. To facilitate comparison with other interventions, we also report the cost per quit for each study arm.

### Satisfaction with the Counseling and Overall Intervention

Six items administered at month 3 assessed participant satisfaction with the number and length of counseling sessions; overall satisfaction with the entire intervention; whether the participant would recommend the program to a friend or family member; and which component of the intervention (counseling, pharmacotherapy guidance, educational materials, or support from health care providers) was most useful.

### Statistical Analyses

All data analyses, except where specified, were conducted using SAS 9.3 [30]. We examined pretreatment differences between groups on demographic, psychosocial, and computer use characteristics using analysis of variance for continuous variables and χ^2^ statistics for categorical variables. To test our primary hypothesis, we compared verified 7-day point-prevalence abstinence at 12 months using the χ^2^ statistic in an intent-to-treat (ITT) analysis, with all non-responders counted as smokers. We repeated our outcome analysis as a multilevel model that controlled for clustering by site using Mplus 6.12 [31]. We also used the χ^2^ statistic and *t* tests, as appropriate, to examine differences between groups for self-reported abstinence, prolonged abstinence, counseling adherence, pharmacotherapy use, quit attempts, and cigarettes per day. We examined differences between groups on participant satisfaction using analysis of variance for continuous variables and χ^2^ test for categorical variables. We compared differences in cost by perspective between the treatment arms using *t* tests. A priori, we specified that if there were a significant difference in outcomes, we would perform an incremental analysis.

Based on a sample size of 283 participants in each group, this study had 80% statistical power at an alpha of .05 to detect a 50% difference between groups in the proportion of participants making a quit attempt (16% in the ITM group and 8% in the Phone group). These quit rates were based on prior studies of in-home telephone counseling referred from primary care providers (Phone) and in-person counseling (ITM) [32,33].

## Results

### Overview

**Figure 1:**
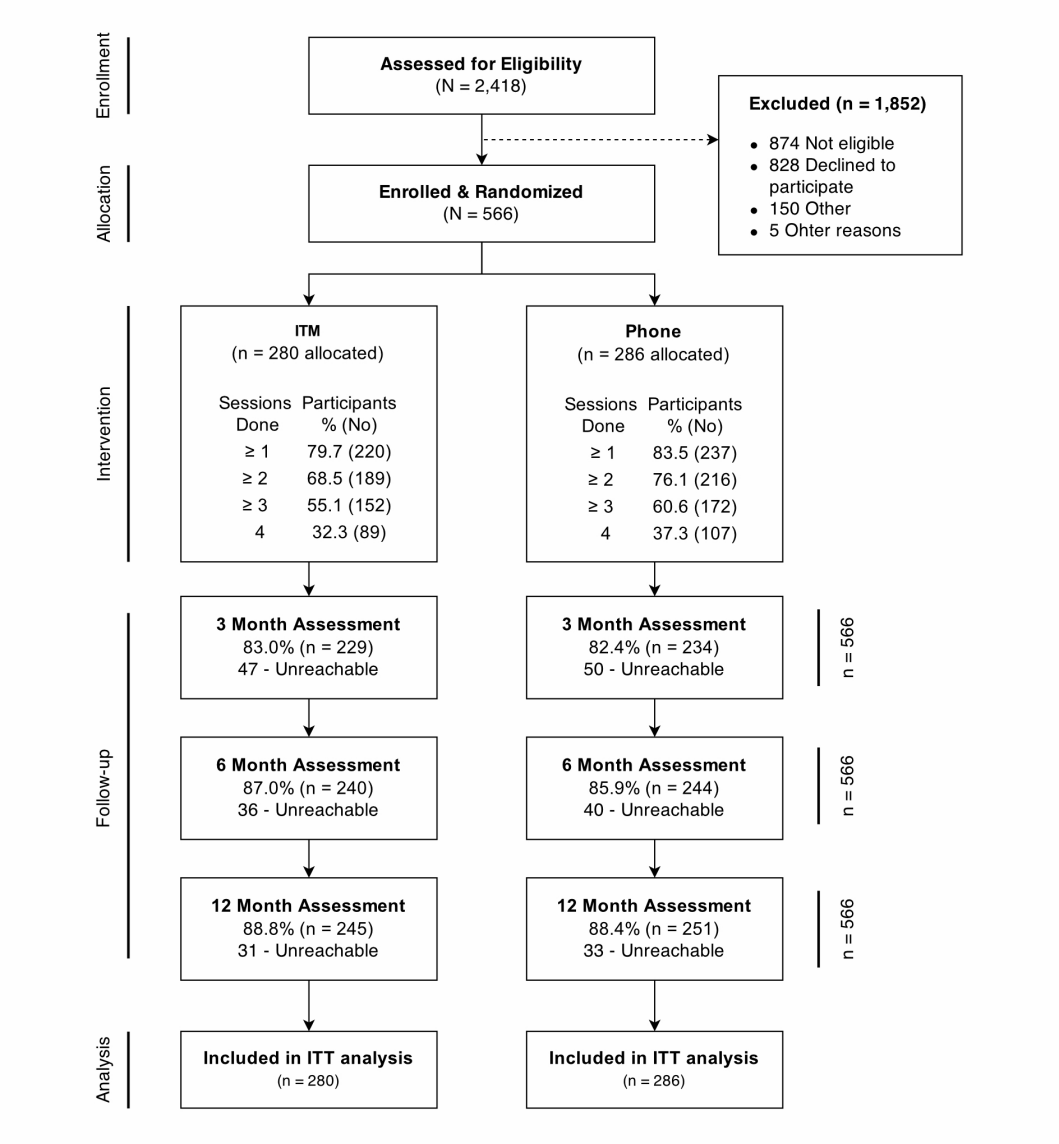
Flow of participants through the trial. Reasons for being dropped from enrolment are not mutually exclusive. Values next to the number of sessions completed represent the cumulative number of participants who completed at least that many treatment sessions. ITT denotes intention to treat.

Of 2418 individuals assessed for eligibility, 1544 were deemed eligible, and 566 provided consent and were respectively randomly assigned to either ITM (280) or Phone (286) (Figure 1). Top reasons for ineligibility included no longer being a smoker (481/874, 55.0%), not having a regular health care provider at the clinic (85/874, 9.7%), and smoking fewer than 5 cigarettes per day (72/874, 8.2%). Follow-up ranged from 83% (470/566) at month 3 to 88% (498/566) at month 12.

### Baseline Characteristics, Computer/Telemedicine Use, and Intervention Fidelity

Randomization resulted in groups with similar baseline characteristics (Table 1). Most (464/566, 82.9%) participants were Caucasian and 9.0% (50/566) were Latino. Most (361/566, 64.5%) had incomes of <200% of the federal poverty level. Chronic diseases including hypertension, chronic lung disease, and diabetes were highly prevalent. Participants smoked on average 19.7 (SD 10.3) cigarettes per day and had moderate nicotine dependence. They began smoking on average at 17.1 (SD 5.0) years of age and most had tried some form of smoking cessation medication in the past. Participants were highly motivated to stop smoking.

There were no differences in computer use variables across groups (Table 1). Although most (70.0%, 391/566) had a working computer at home, one out of three lacked home Internet access, nearly half were not comfortable using computers, and only 4.5% (25/566) had been seen by a doctor via telemedicine in the past. Many were not confident that personal information was kept private via technology, were not comfortable using newer communication technologies, and were not interested in receiving telecare in the home. Analysis of fidelity data found no significant differences in counseling style across the groups.

**Table 1:** Baseline characteristics.

			ITM n=280	Phone n=286	Total n=566
			n (%) or mean (SD)	n (%) or mean (SD)	n (%) or mean (SD)
**Sociodemographics**					
	Age, yrs, mean (SD)		47.27 (12.8)	47.51 (13.0)	47.4 (12.9)
	Female		173 (62.7)	190 (66.9)	363 (64.8)
	Caucasian		228 (82.6)	236 (83.1)	464 (82.9)
	Hispanic/Latino		23 (8.3)	27 (9.6)	50 (9.0)
	Married		123 (44.6)	115 (40.6)	238 (42.6)
	High school education or less		161 (58.3)	156 (55.2)	317 (56.8)
	Employed full time		110 (39.9)	123 (43.5)	233 (41.7)
	<200% Federal Poverty Level		177 (64.1)	184 (64.8)	361 (64.5)
	Health insurance		174 (63.0)	178 (62.7)	352 (62.9)
	Prescription cessation medication coverage		161 (58.3)	159 (56.2)	320 (57.3)
**Medical history**					
	Hypertension		121 (43.8)	121 (42.8)	242 (43.3)
	High cholesterol		119 (43.1)	103 (36.4)	222 (39.7)
	Chronic lung disease		91 (33.0)	98 (34.6)	189 (33.8)
	Diabetes		55 (19.9)	48 (17.0)	103 (18.4)
	Heart disease		30 (10.9)	28 (9.9)	58 (10.4)
	Cancer		23 (8.3)	25 (8.8)	48 (8.6)
	Stroke		8 (2.9)	15 (5.3)	23 (4.1)
**Mental health co-morbidities**					
	PHQ-2, depression^a^		137 (49.6)	142 (50.2)	279 (49.9)
	AUDIT-C, high risk drinking^b^		58 (21.0)	71 (25.0)	129 (23.0)
	GAD-2^c^		115 (41.7)	111 (39.2)	226 (40.4)
**Current smoking**					
	Current cigarettes per day, mean (SD)		20.3 (10.7)	19.2 (9.8)	19.7 (10.3)
	Nicotine dependence (FTND), mean (SD)		4.91 (2.2)	4.85 (2.4)	4.9 (2.3)
**Smoking history**					
	Age started smoking regularly, yrs, mean (SD)		16.9 (4.4)	17.4 (5.5)	17.1 (5.0)
	No. quit attempts, past 12 months, mean (SD)		2.0 (3.2)	2.1 (2.9)	2.0 (3.1)
	Prior use of cessation pharmacotherapy (any)		210 (76.1)	200 (70.7)	410 (73.4)
	Longest period of past abstinence in days, mean (SD)		331.9 (768.2)	432.6 (1016.2)	382.9 (902.9)
**Interest in quitting**					
	**Readiness to stop smoking**				
		Pre-contemplation	7 (2.5)	7 (2.5)	14 (2.5)
		Contemplation	105 (38.0)	113 (39.9)	218 (39.0)
		Preparation	164 (59.4)	163 (57.6)	327 (58.5)
	Importance of quitting (0-10 low-high), mean (SD)		9.4 (1.5)	9.3 (1.5)	9.4 (1.5)
	PCSC (1-7 low-high), mean (SD)^d^		5.0 (1.5)	5.0 (1.5)	5.0 (1.5)
**Computer, Internet, and telemedicine use**					
	Currently have a functional computer in home		203 (73.6)	188 (66.4)	391 (70.0)
	Currently have Internet access in home		193 (69.3)	182 (64.3)	375 (67.1)
	Ever been seen by a doctor via telemedicine, ITV, or webcam		11 (4.0)	14 (5.0)	25 (4.5)
**Attitudes toward computers, communication technology, and health technology**					
	I am comfortable using computers, (% agree-strongly agree)		161 (58.3)	173 (61.1)	334 (59.8)
	I am comfortable using other communication technologies, such as mobile phones, mp3 players, or Web cameras, (% agree-strongly agree)		179 (64.9)	172 (60.8)	351 (62.8)
	I am interested in receiving health care in my home using computers or communication technologies, (% agree-strongly agree)		155 (56.2)	168 (59.4)	323 (57.8)
	I am confident my personal information is kept private when using communication technologies, (% agree-strongly agree)		194 (70.3)	183 (64.7)	377 (67.4)

^a^PHQ-2: Patient Health Questionnaire, 2-item version.

^b^AUDIT-C: Alcohol Use Disorders Identification Test - Consumption, with a binge drinking cutoff of >4 Males, >3 Females.

^c^GAD-2: Generalized Anxiety Disorder questionnaire, 2-item version.

^d^PCSC: Perceived Competence Scale for Cessation.

### Hypothesis 1: Primary Outcome

**Figure 2:**
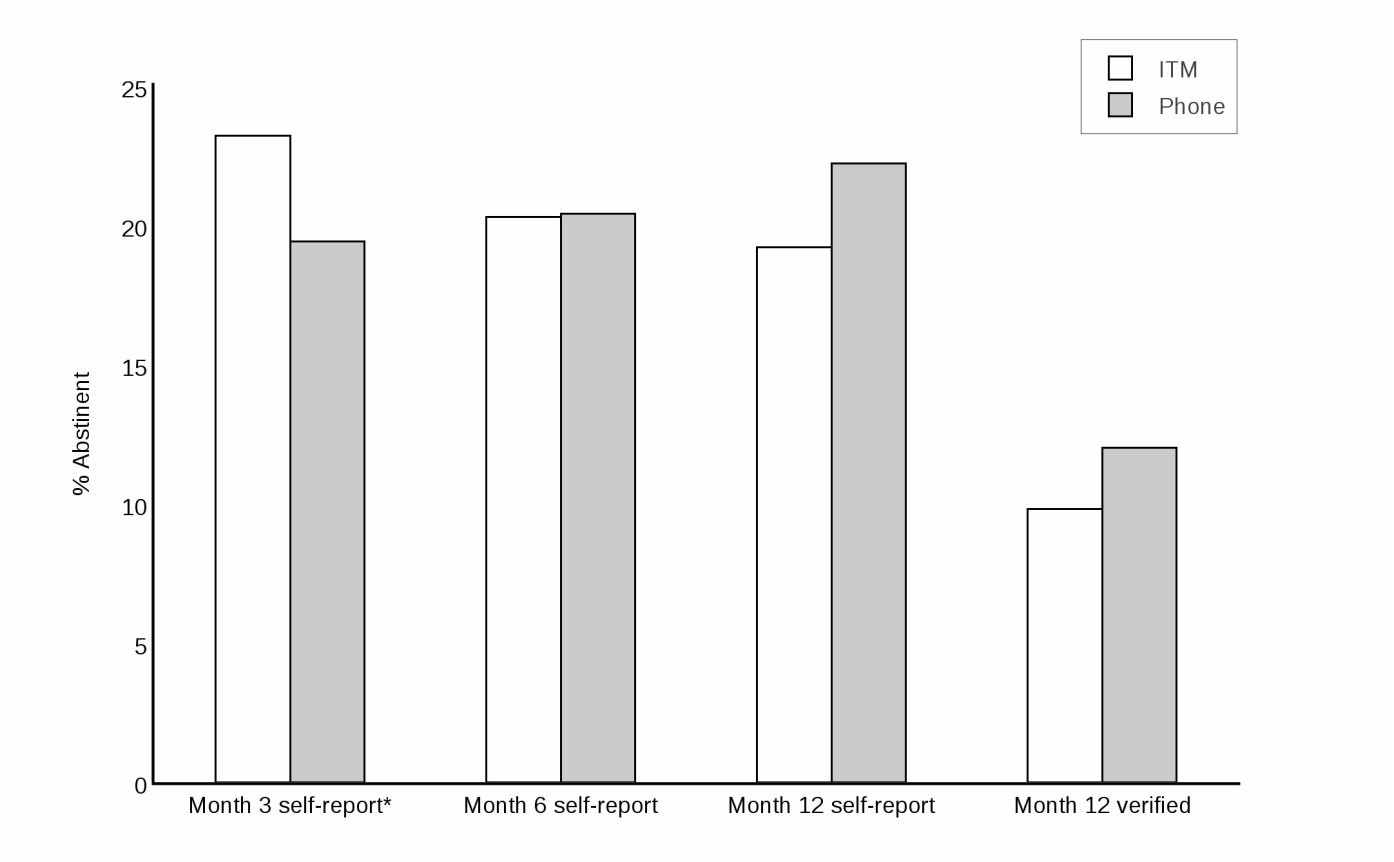
Primary outcomes.

The main outcome of biochemically verified 7-day point prevalence did not significantly differ between ITM and Phone at 12 months (9.8% vs 12.0%, 27/566 vs 34/566; *P*=.406) (Table 2). These rates were also not different when treated as a multi-level model that controlled for clustering (*P*=.554). Participants in ITM reported higher abstinence rates at month 3, similar rates at month 6, and lower rates at month 12 compared to Phone; none of these differences, however, were statistically significant (Figure 2).

### Hypotheses 2-3: Prolonged Abstinence, Counseling Adherence, Pharmacotherapy Use

Likewise, prolonged abstinence did not differ significantly between ITM and Phone (*P*=.8394) (Table 2). Phone participants completed slightly more counseling sessions than ITM. Significantly more participants in ITM used cessation medications, compared to Phone (*P*=.03).

### Hypothesis 4: Quit Attempts and Number of Cigarettes Smoked Among Continued Smokers

Among participants who continued to smoke, those in ITM made somewhat more attempts to quit compared to those in Phone (4.8, SD 6.8 vs 4.3, SD 5.7); this difference, however, was not significant (Table 2). Continued smokers in both study arms reported smoking similar numbers of cigarettes per day.

**Table 2:** Outcomes: Hypotheses 1-4.

Hypothesis			ITM n=280	Phone n=286	*P* value
**Main outcomes, n=566**					
	1	Biochemically verified 7-day point prevalence, 12 months, n (%)	27 (9.8)	34 (12.0)	.406
	2	Prolonged abstinence, n (%)	23 (8.1)	21 (7.6)	.8394
	3	Average number of counseling sessions, mean (SD)	2.4 (1.5)	2.6 (1.5)	.083
	3	Used cessation medication, n (%)	128 (55.9)	107 (46.1)	.03
**Outcomes among participants continuing to smoke at 12 months, n=499**					
	4	Average number of quit attempts, mean (SD)	4.8 (6.8)	4.3 (5.7)	.3469
	4	Average number of cigarettes smoked per day at 12 months, mean (SD)	10.2 (8.1)	10.0 (7.5)	.7212

### Hypothesis 5: Costs

#### Provider Perspective

Costs are summarized in Table 3 (US $). Counseling time costs differed by 9% (US $4.12) between the treatment arms. Communication (Internet or phone) costs were lower for the ITM arm. The cost for the clinic space used to deliver ITM had a large impact on provider costs, depending on how the space was valued. When the space was valued as local rental space, the cost was $0.99 per person; when valued as a medical visit, the cost was $226.61 per participant. Summing all provider costs, the mean cost for the Phone arm was $53.25 as compared to $47.04 for ITM when space was valued at rental rate. When physician office visit rates were used to value the space, the cost of ITM increased substantially to $272.65.

#### Participant Perspective

From the participant’s perspective, counseling time costs were roughly 10% higher in the Phone arm, consistent with the counselor time costs. Participants bore a much heavier burden in ITM, though, because of the travel-related time and mileage costs, which added almost $94 to ITM participant costs.

#### Societal Perspective and Cost Per Quit

After summing across provider and participant costs, Phone was less costly than ITM regardless of underlying assumptions ($81.61, SD 58.70 per participant for Phone). The magnitude of the difference between approaches differed greatly depending on how we valued the clinic space used to deliver ITM ($166.04, SD 347.90 if rental cost basis or $390.20, SD 415.40 if CPT cost basis for clinic space). We did not compute an incremental cost-effectiveness ratio, as there was no significant difference in the primary outcomes between ITM and Phone. The cost per quit from the provider perspective was $444/quit for Phone and $480/quit for ITM in provider costs. Adding patients’ costs increased those values to $680/quit (Phone) and $1694/quit (ITM).

#### Satisfaction With Counseling and Overall Intervention

Satisfaction with the study was high. Overall, participants were somewhat satisfied (26.8%, 99/369) or very satisfied (73.2%, 270/369) with the program. Most (72.6%, 281/387) reported the length of sessions was about right. When asked to choose the most helpful part of the program, most (61.2%, 243/392) participants chose counseling. The only difference between study arms was the proportion of participants who would recommend the program to a friend or family member: 97% (194/200) of ITM participants reported they would recommend (74.5%, 149/200) or had already recommended it (22.5%, 45/200) compared to 91.9% (182/198) in the Phone arm reported that they would recommend (78.8%, 156/198) or had already recommended it (13.1%, 26/198) to a friend or a family member (*P*=.0075).

**Table 3:** Input valuations and results for variable cost components by intervention arm (US $).

Variable costs				Unit costs	Phone (n=284)		ITM (n=276)	*P* value
					Mean (SD) in $			
**Provider perspective**								
		Counselor cost		$28.81/hour	49.58 (33.35)		45.46 (31.50)	.133
		Internet access		$0.37/hour	n/a		0.58 (0.40)	
		Telephone charges		$2.13/hour	3.67 (2.47)		n/a	
		Facility costs		rent basis	n/a		0.99 (0.69)	
		Facility costs		CPT basis	n/a		226.61 (148.08)	
	**Total provider variable costs**							
		Calculated based on costs to rent space			53.25 (35.82)		47.04 (32.59)	.032
		Calculated based on costs for medical visit			53.25 (35.82)		272.65 (178.29)	<.001
**Participant perspective**								
		Time in counseling		Hourly wage	28.36 (27.83)		25.81 (21.24)^a^	.224
		Travel time cost		Hourly wage	n/a		33.38 (101.27)^a^	
		Mileage costs		$0.54/mile	n/a		60.59 (239.93)^a^	
		Pharmacotherapy		out-of-pocket costs	113.87=16,852 total (n=148)		92.21=6544 total (n=150)	
					59.34/participant		51.04/participant^a^	
	**Total participant variable costs**							
		Without pharmacotherapy			28.36 (27.80)		119.44^a^ (341.00)	<.001
		With pharmacotherapy			75.29 (169.10)		124.55^a^ (259.10)	.008
	**Combined societal (modified) perspective**							
		Rent basis			81.61 (58.70)		166.04^a^ (347.90)	<.001
		Facility CPT basis			81.61 (58.70)		390.20^a^ (415.40)	<.001

^a^n=271: 5 additional ITM participants had missing self-reported participant perspective cost data.

## Discussion

### Principal Findings

Integrated telemedicine was not superior to phone-delivered counseling for smoking cessation. While telemedicine had the added benefit of increasing pharmacotherapy utilization, telephone counseling facilitated adherence to counseling sessions. There are relative benefits to each intervention approach, and both promote smoking abstinence, but telephone counseling was significantly less expensive. In our trial, the provider cost of telemedicine-delivered counseling was either equivalent to phone or much more expensive, depending on the assumption underlying the cost of the space used to deliver the telemedicine-based intervention.

### Limitations

Because the study tested the effects of telemedicine integrated into physician practices, versus telephone counseling delivered to patients’ homes, it is impossible to isolate the effects of video- versus phone-based counseling. Moreover, we limited our study to rural practices in the Midwest. These findings might not generalize to other settings. We opted to test integrated telemedicine in part because we were concerned that our rural population might have difficulty navigating a home-based telemedicine intervention, or a video intervention via smartphones. As of 2011, only 21% of rural adults were smartphone users [34]. We believe this concern was borne out by our participants’ low rates of comfort using computers and familiarity with smartphone technology. We were not always able to determine the space used for the telemedicine encounter, which necessitated calculating costs under two different space assumptions. While our intervention included components of both MI and CBT, our fidelity assessment was limited to adherence to MI procedures alone, and did not include a component assessing fidelity of CBT across arms. Last, one rationale for this study was that quitlines have low rates of utilization by smokers. This study, however, does not determine whether integrated telemedicine would have higher utilization.

### Comparison With Prior Work

Our biochemically verified quit rates, in both study arms, are similar to self-reported long-term quit rates reported by smokers using telephone quitlines [3]. Likewise, our 3-month self-reported quit rate of 23% in our ITM arm was similar to the 25% self-reported 3-month quit rate reported by Carlson et al, in their rural telemedicine group-based cessation intervention [15].

In comparison with recent studies, our cost per quit values are consistent: $444/quit for Phone and $480/quit for ITM in provider costs. Adding patients’ costs increased those values to $680/quit (Phone) and $1694/quit (ITM). A telephone-based counseling approach from Veterans Affairs reported $1092/quit (2009 dollars) and an Australian quitline reported $680/quit (converted Australian to US dollars) [35,36]. Unlike most telemedicine trials, we compared our telemedicine intervention to a telephone intervention, *not* to a face-to-face intervention [14]. Undoubtedly, the telemedicine would have been less expensive compared to the cost of traveling to a location for equivalent quality face-to-face counseling—which would have been Kansas City.

From a provider’s perspective (the organization proposing to deliver the intervention), if they have an appropriate space to perform the telemedicine intervention that would not influence the clinic’s revenue generation, then ITM would be the preferred approach, given the higher propensity of participants to refer ITM to family and friends. From a patient’s perspective, attending a face-to-face or a telemedicine intervention outside the home imposes a substantial burden in time and travel costs. This may well, in practice, ultimately limit attendance. From a societal perspective, applying limited resources to the best yield tips the balance to the current phone-based quitline as most cost-effective.

### Conclusions

Findings did not support the superiority of telemedicine smoking cessation counseling, integrated into patients’ medical homes, over quitline counseling. Participants in the telemedicine arm, however, were significantly more likely to recommend the program to family and friends, in spite of the fact that ITM placed considerably higher burden on participants. This is important. Although all states provide free access to telephone quitlines, very few smokers choose to use quitlines. Telemedicine-based counseling, integrated into medical homes, could be another option for behavioral counseling for smokers who might not opt for phone counseling. The opportunity costs associated with using clinic exam rooms for delivering telemedicine counseling made this approach far less favorable economically, however. Identifying a less costly space to deliver telemedicine within patients’ medical homes would maximize the efficiency of this approach.

Future research could include preference trials, in which smokers are provided the option of choosing between telemedicine and quitline counseling, to examine whether the higher proportion of participants who would refer to family and friends translates into higher rates of utilization. Moreover, there may be sub-populations of smokers for whom this form of telemedicine might be more attractive or more effective. Future studies, perhaps involving mixed modeling or classification and regression tree (CART) analyses might identify such groups.

Because our trial was designed as a test of superiority and not equivalence, it is premature to assume that the effects of quitline and ITM are equivalent. In our trial, differences between the groups consisted of (1) participant willingness to refer others to the study, and (2) costs. It would be misleading, however, to suggest that decisions about implementation be made on the basis of these differences alone. A future equivalence trial would better determine the relative effectiveness of each approach and might uncover other implementation considerations. In addition, future interventions could combine and test elements of both approaches to optimize pharmacotherapy utilization, counseling adherence, and satisfaction. Such an approach could commence with a telemedicine-delivered clinic office visit for pharmacotherapy guidance, and continue with either telephone or video counseling delivered via traditional or mobile phones to flexibly deliver behavioral support to patients where they most need it—in their homes and communities.

## Abbreviations

AUDIT-C: Alcohol Use Disorders Identification Test, alcohol consumption questions
CART: Classification and Regression Tree
CBT: Cognitive Behavior Therapy
CO: carbon monoxide (expired)
CPT: American Medical Association’s Current Procedural Terminology
FTND: Fagerström Test for Nicotine Dependence
GAD-2: Generalized Anxiety Disorder questionnaire, 2-item version
ITM: integrated telemedicine
ITT: intent-to-treat
MI: motivational interviewing
MITI: Motivational Interviewing Treatment Integrity
PCSC: Perceived Competence Scale for Cessation
Phone: telephone treatment arm
