# Metadata Correction: Comparative and Cost Effectiveness of Telemedicine Versus Telephone Counseling for Smoking Cessation

**DOI:** 10.2196/jmir.4642

**Published:** 2015-06-15

**Authors:** Kimber P Richter, Theresa I Shireman, Edward F Ellerbeck, A Paula Cupertino, Delwyn Catley, Lisa Sanderson Cox, Kristopher J Preacher, Ryan Spaulding, Laura M Mussulman, Niaman Nazir, Jamie J Hunt, Leah Lambart

**Affiliations:** ^1^University of Kansas Medical CenterDepartment of Preventive Medicine and Public HealthKansas City, KSUnited States; ^2^University of Missouri, Kansas CityDepartment of PsychologyKansas City, MOUnited States; ^3^Vanderbilt UniversityDepartment of Psychology & Human DevelopmentNashville, TNUnited States; ^4^University of Kansas Medical CenterCenter for Community EngagementKansas City, KSUnited States; ^5^University of Missouri, Kansas CitySchool of Nursing and Health StudiesKansas City, MOUnited States

The authors of “Comparative and Cost Effectiveness of Telemedicine Versus Telephone Counseling for Smoking Cessation” (J Med Internet Res 2015;17(5):e113) inadvertently omitted Delwyn Catley, PhD (University of Missouri, Kansas City, Department of Psychology, Kansas City, MO, United States) from the list of authors during the submission process. The author Catley should have been added after A Paula Cupertino in the original published manuscript. In addition, the affiliation for the author Sherman should have been the same as the author Richter. Last, the telephone and fax numbers were not the most up to date numbers. These should be Phone: 1 9135882718, Fax: 1 9135882780 instead of Phone: 1 9134490157, Fax:1 9134490157. These errors have been corrected in the online version of the paper on the JMIR website on June 15, 2015, together with publishing this correction notice. This was done after submission to PubMed Central and other full-text repositories. This correction notice has been submitted to PubMed, the original paper resubmitted to PubMed Central, and the metadata has been resubmitted to CrossRef with publishing this correction notice.

